# Dynamic and temporal assessment of human dried blood spot MS/MS^ALL^ shotgun lipidomics analysis

**DOI:** 10.1186/s12986-017-0182-6

**Published:** 2017-03-20

**Authors:** Fei Gao, Justice McDaniel, Emily Y. Chen, Hannah E. Rockwell, Jeremy Drolet, Vivek K. Vishnudas, Vladimir Tolstikov, Rangaprasad Sarangarajan, Niven R. Narain, Michael A. Kiebish

**Affiliations:** BERG, LLC, 500 Old Connecticut Path, Bldg B, 3rd Floor, Framingham, MA 01701 USA

**Keywords:** Human, Dried blood spot, Shotgun lipidomics, Mass spectrometry

## Abstract

**Background:**

Real-time and dynamic assessment of an individual’s lipid homeostatic state in blood is complicated due to the need to collect samples in a clinical environment. In the context of precision medicine and population health, tools that facilitate sample collection and empower the individual to participate in the process are necessary to complement advanced bioanalytical analysis. The dried blood spot (DBS) methodology via finger prick or heel prick is a minimally invasive sample collection method that allows the relative ease and low cost of sample collection as well as transport. However, it has yet to be integrated into broad scale personalized lipidomic analysis. Therefore, in this study we report the development of a novel DBS high resolution MS/MS^ALL^ lipidomics workflow.

**Methods:**

In this report we compared lipidomic analysis of four types of blood sample collection methods (DBS, venous whole blood, serum, and plasma) across several parameters, which include lipidomics coverage of each matrix and the effects of temperature and time on the coverage and stability of different lipid classes and molecular species. The novel DBS-MS/MS^ALL^ lipidomics platform developed in this report was then applied to examine postprandial effects on the blood lipidome and further to explore the temporal fluctuation of the lipidome across hours and days.

**Results:**

More than 1,200 lipid molecular species from a single DBS sample were identified and quantified. The lipidomics profile of the DBS samples is comparable to whole blood matrix. DBS-MS/MS^ALL^ lipidomic analysis in postprandial experiments revealed significant alterations in triacylglyceride species. Temporal analysis of the lipidome at various times in the day and across days identified several lipid species that fluctuate as a function of time, and a subset of lipid species were identified to be significantly altered across hours within a day and within successive days of the week.

**Conclusions:**

A novel DBS-MS/MS^ALL^ lipidomics method has been established for human blood. The feasibility and application of this method demonstrate the potential utility for lipidomics analysis in both healthy and diverse diseases states. This DBS MS-based lipidomics analysis represents a formidable approach for empowering patients and individuals in the era of precision medicine to uncover novel biomarkers and to monitor lipid homeostasis.

**Electronic supplementary material:**

The online version of this article (doi:10.1186/s12986-017-0182-6) contains supplementary material, which is available to authorized users.

## Background

For over two decades the dried blood spot (DBS) methodology has commonly been used in the diagnosis of inborn errors of metabolism [1–3]. Currently, more than 95% of newborns are screened for inherited metabolic disorders using DBS in the United States [2, 4]. Using this methodology blood samples are typically obtained from heel or finger pricks and spotted onto filter paper for collection and analysis. DBS offers several advantages over conventional whole blood, plasma or serum sample collection [5, 6]. For example, DBS sample collection is minimally invasive and easy to perform (e.g. finger or heel prick rather than venous puncture) and notably, it can be done at home by patients or volunteers themselves after minimal training. In addition, a low volume of blood (less than 20 μL) is needed to spot onto filter paper compared to a minimum of 0.5 mL of blood in venous sampling. Lastly, DBS is relatively stable at ambient temperature, thus facilitating easy shipping and storage of samples. As such, the application of DBS has been developed for therapeutic drug monitoring [7, 8], pharmacokinetics [9], genomics [10], proteomics [11, 12], and metabolomics [13].

The lipidome of human blood is comprised of thousands of lipid molecular species [14]. Its homeostatic regulation plays a pivotal role in numerous pathological disease states, such as atherosclerosis, cancer, metabolic diseases, and Alzheimer’s disease [15–20]. Lipidomic analysis is largely based on electrospray ionization mass spectrometry (ESI-MS) coupled with liquid chromatography (LC) or using direct infusion, i.e., shotgun lipidomics [21–23]. Using the ESI-MS/MS strategy, lipid molecular species can be identified by their characteristic fragments (from the head group or fatty acid chains) and their molecular weights. Typically, these types of ions have been applied in precursor ion scanning, neutral loss scanning, and multiple reaction monitoring experiments to measure lipid species from human serum, plasma, and whole blood [23, 24]. Traditional MS-based lipidomics has been dominated by triple quadrupole instruments and require the pre-selection of specific ions or multiple runs for the diverse capture of the lipidome. Recently, a novel data-independent acquisition (DIA) technique of mass spectrometry has been developed to overcome this shortcoming by parallelizing the fragmentation of all detectable ions within a certain m/z range [25, 26]. With the combination of high-resolution MS, DIA provides a powerful tool for shotgun lipidomics. MS/MS^ALL^ is a direct infusion DIA technique, using a hybrid quadrupole time-of-flight (QTOF) technology, specifically designed for lipidomics [27, 28]. In MS/MS^ALL^, all precursors are selected in the Q1 quadrupole at unit-based resolution in a step-wise fashion to completely cover the entire mass range of interest. Collision-induced dissociation is carried out in Q2 at high speed and the corresponding high-resolution MS/MS spectra are recorded covering every precursor in each cycle [28]. The combination of high resolution, sensitivity, and throughput offers significant advantages towards the identification and quantification of lipids in complex biological extracts.

Here in, we report a high-throughput DBS-MS/MS^ALL^ lipidomics platform to analyze the blood lipidome via the direct infusion of lipids extracted from DBS samples. More than 1,200 lipid molecular species were identified and quantified in a single dried blood spot sample. We also explored the lipidomics profiles from four different blood matrixes: 1) dried blood spot from a finger prick, 2) whole blood (through venous sampling), 3) plasma, and 4) serum. In addition, DBS lipid stability at various storage temperatures and time points was assessed. The high-throughput DBS-MS/MS^ALL^ shotgun lipidomics workflow demonstrated here exemplifies a robust and formidable tool for investigation of population health and precision medicine in diverse disease states and interventions that empower individual participation and avoids unnecessary sample waste.

## Methods

### Materials

All lipid standards were purchased from Nu-Chek Prep Inc (Waterville, MN), Avanti Polar Lipids (Alabster, AL), Cayman Chemical (Ann Arbor, MI), Matreya (State College, PA), Cambridge Isotope Laboratories (Tewksbury, MA), or Sigma-Aldrich (St Louis, MO). All solvents were of HPLC or LC/MS grade and were purchased from Fisher Scientific (Waltham, MA) or VWR International (Radnor, PA). Whatman 903™ filter paper cards, adjusted ACCU-CHEK lancets, and Uni-Core punch with ID 6 mm were purchased separately from GE Healthcare (Westborough, MA) and Roche Diagnostics (Indianapolis, IN).

### Preparation of dried blood spots

Written informed consent was provided by the subjects prior to participating in the study. Research use of samples was conducted in accordance with the terms outlined within the informed consent form and the terms set forth therein and with the tenets of the Declaration of Helsinki. Diets and other physiologic parameters were not controlled for in this study.

For the blood matrix study, the volunteer was fasted overnight (around 12 h) and venipuncture of a cubital vein was performed in the morning. Blood was drawn into a non-coated tube for whole blood, a K2 EDTA tube (BD, Franklin Lakes, NJ) for blood plasma, and silicone coated tube (BD) for blood serum. For whole blood samples, tubes were mixed well by gentle inversion 10 times and whole blood was immediately spotted on Whatman 903™ filter paper cards. For serum and plasma samples, the tubes were centrifuged at 1,200 *g* for 15 min at room temperature and the supernatant plasma and serum were spotted onto Whatman 903™ filter paper cards. Concomitantly, blood via finger prick was taken and spotted onto Whatman 903™ filter paper card. All samples were air dried for 3 h at room temperature (~22 °C) and collected using Uni-Core punch (ID 6 mm, GE Healthcare) for extraction as described below.

For the DBS stability study, whole blood was drawn and immediately spotted onto Whatman 903™ filter paper cards. After samples were air dried completely for 3 h at room temperature, the paper cards were stored with desiccant at different conditions: 4 °C, room temperature, and 37 °C for 3 days, 1 week, and 2 weeks.

For the time course study, a total of 12 healthy volunteers (6 male and 6 female) were included (Mean age 31 ± 6 years). After finger pricks using a single-use safety lancet, the blood drop was directly applied onto Whatman 903™ filter paper cards at 7 AM (after overnight fasting), 10 AM, 1 PM (1 h after lunch), 4 PM, and 7 PM. The blood spots were air dried completely for 3 h at room temperature and stored at room temperature in a zip-closure bag with desiccant.

For the DBS daily variation study, a total of 16 healthy volunteers (8 male and 8 female) were included (Mean age 32 ± 6 years). Finger prick blood samples were collected onto Whatman 903™ filter paper cards in the morning after overnight fasting over 5 consecutive days. The blood spots were air dried completely for 3 h at room temperature and stored at room temperature in a zip-closure bag with desiccant.

### Sample preparation and extraction

The dried blood spots were punched out uniformly using a GE Healthcare Uni-Core punch with ID 6 mm and transferred to glass tubes. Lipid extraction was performed using a modified Bligh and Dyer as previously described [22, 27, 29]. Four mL chloroform:methanol (1:1, v/v) and 1.6 mL LiCl solution (50 mM) were added to each sample with a cocktail of deuterium-labeled, or odd chain and extremely low naturally abundant fatty acid internal standards for diverse lipid classes (Additional file 1: Table S1). The extraction homogenate was vortexed and centrifuged at 1000 *g* for 5 min. The chloroform layer of each extract mixture was carefully removed and saved. An additional 2 mL chloroform was added into the MeOH/aqueous layer of each test tube. After centrifugation, the chloroform layer from each individual sample was combined and dried under a nitrogen stream. Each individual residue was then re-suspended in 4 mL chloroform/methanol (1:1), back-extracted against 1.8 mL LiCl aqueous solution (10 mM), and the extract was dried as described above. Such lipid extraction was automated using a customized sequence on a Hamilton Robotics STARlet system (Hamilton, Reno, NV) designed to meet high-throughput requirements. Finally, lipid extracts were dried under nitrogen and reconstituted in 68 μL chloroform:methanol (1:1, v/v). Samples were flushed with nitrogen and stored at -20 °C. The reproducibility of the extraction method was evaluated by measuring the peak area of the added internal standard in three replicates of DBS. The recovery was obtained by comparing the response of analyte added and extracted from the DBS to the response for the analyte in solvent. The sensitivity was assessed as the limit of quantification (LOQ) for each added internal standard by measuring the serial dilution of the extracted DBS samples (Additional file 2: Table S2).

### Direct Infusion Quadruple Time of Flight (QTOF) mass spectrometry

The concentrated samples were diluted 50× in isopropanol:methanol: acetonitrile:H_2_O (3:3:3:1, by vol.) with 2 mM ammonium acetate in LC vials, and 50 μL diluted lipid extract was automatically loaded and directly delivered to the ESI source using an Ekspert microLC 200 system (Sciex) with a flow rate of 6 μL/min on a customized sample loop. The MS/MS^ALL^ acquisition experiment was carried out on a SCIEX TripleTOF 5600+ (Sciex). ESI source parameters included nebulizing gases GS1 at 10, GS2 at 10, curtain gas (CUR) at 15, ion spray voltage at 5000 and -4500 V for the positive and negative modes, respectively, declustering potential at 100 V, and temperature at 300 °C. The atmospheric-pressure chemical ionization (APCI) probe and inlet were connected to a calibrant pump which delivers mass calibration solution for MS and MS/MS. The MS/MS^ALL^ data acquisition was controlled with Analyst® TF 1.5.1 software (Sciex). The MS range was 200-1200 m/z at an accumulation time of 300 ms, followed by 1000 product ion scans with 1000 precursors evenly spaced from m/z 200.051 to m/z 1200.051, measuring across m/z 200-1200 with the accumulation time 300 ms each. The total time for one MS/MS^ALL^ acquisition was around 8 min. Optimal collision energy MS/MS fragmentation was 30 ± 15 eV. The identification of lipid molecular species was based on the molecular weight, at least one specific fragments, and lipid structure (Table 1). The acquired TOF MS and MS/MS^ALL^ data were processed with MultiQuant 1.2.2.5 (Sciex) with the support of MS/MS^ALL^ data analysis and the in-house database. Furthermore, the identification of a lipid molecular species structure could be further confirmed using PeakView® software (Sciex), if needed. The quantification was performed by comparing the peak area of molecular species to the internal standard within a linear dynamic range with the isotopic corrections [30] and normalized to one DBS spot using MultiQuant 1.2.2.5. The lipid species were counted based on the readout obtained from MultiQuant as confirmed by diagnostic fragments used for quantitation.

**Table 1:** Summary of polarity and scan modes for lipid classes using ESI-MS/MS^ALL^

Lipid Class^a^	Polarity	Molecular Ion	MS/MS Scan Mode
PC	positive	M + H	Precursor ion of 184.1
PE	positive	M + H	Neutral loss of 141.0
SM	positive	M + H	Precursor ion of 184.1
TAG	positive	M + NH_4_	Neutral loss of FA^b^
DAG	positive	M + NH_4_	Neutral loss of FA
Cer	positive	M + H	Precursor ion of 264.2
AC	positive	M + H	Precursor ion of 85.0
Glycolipid	positive	M + H	Precursor ion of 264.2
PA	negative	M-H	Precursor ion of FA
PS	negative	M-H	Precursor ion of FA
PI	negative	M-H	Precursor ion of FA
PG	negative	M-H	Precursor ion of FA

^a^ *PC* phosphatidylcholine, *PE* phosphatidylethanolamine, *PS* phosphatidylserine, *SM* sphingomyelin, *PI* phosphatidylinositol, *PG* phosphatidylglycerol, *PA* phosphatidic acid, *TAG* triacylglyceride, *DAG* diacylglyceride, *Cer* ceramide, *AC* acylcarnitines, ^b^ *FA* fatty acid

### Statistics

The results are expressed as mean ± standard deviation (SD). Statistical analysis comparing various blood matrices coverage was performed using Graphpad Prism. The analysis of lipids over time was conducted using a repeated one-way analysis of variance ANOVA using R programming [31]. The principal component analysis (PCA) was carried out for the different blood matrix study using R programming [32].

## Results

### Adaption of DBS-MS/MS^ALL^ shotgun lipidomics to different blood matrix samples

The choice of negative mode or positive mode analysis for different lipid classes is based on the differential propensity of each lipid class to acquire either positive or negative charges under the source of high voltage [15, 22, 33]. Anionic lipids, including PA, PG, PI, and PS are acquired under the negative modes for the higher sensitivity. The zwitterion phospholipids, such as PC, PE, and SM, and the neutral lipids, prefer to become either the protonated ions or ammonium added ions in the positive modes. For each different lipid class, the quantification was carried out by either precursor ion scanning or neutral loss scan, specific for acyl fatty acid anions, or lipid class-specific fragment ions [22, 24]. The ionization mode, molecular ions, and their corresponding scan modes are summarized in Table 1.

Lipid extraction is one of the key steps to the successful analysis of shotgun lipidomics by ESI/MS in general. In this study, the modified Bligh and Dyer method was used to extract total lipids from DBS samples and is suited for analyzed lipid classes [22, 33]. LiCl solution was used to improve the extraction efficiency of acidic lipids, prevent the degradation of plasmalogen molecular species, and decrease spectral complexity. At the final stage, an additional Bligh and Dye extraction against an aqueous phase with LiCl was used to further enrich the lipid extracts. To evaluate the efficiency of DBS lipid extraction, the repeatability and recovery of the extraction was investigated using the added internal standards. The recovery rates for most of added lipid analytes are in the range of 50 to 100% with satisfactorily reproducible measurements (coefficient of variation [CV] <10% for most added lipid analytes) (Additional file 2: Table S2). The sensitivity of the MSMS^ALL^ shotgun lipidomics, represented by LOQ, ranging from 0.10 to 1.62 nM, is consistent with the previous reports [22, 23, 27, 28, 33].

**Fig. 1:**
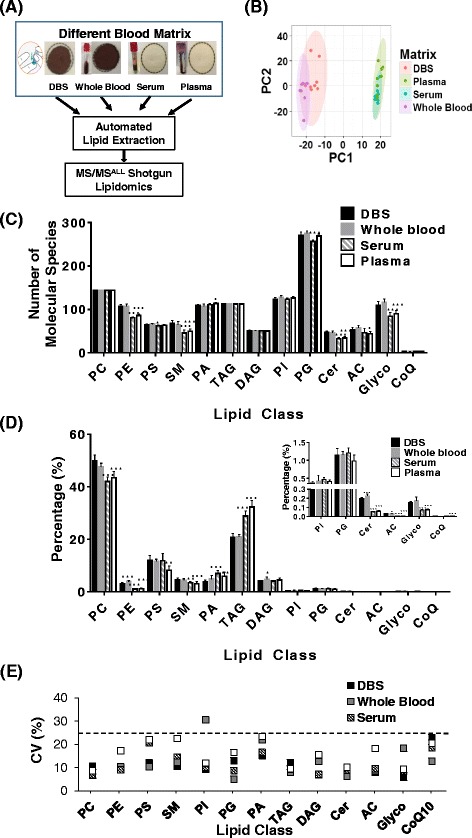
Adaption of dried blood spots (DBS)-MS/MS^ALL^ Lipidomics to different blood matrices. (**a**) Scheme of DBS-Lipidomics for blood matrices; (**b**) Principle component analysis (PCA) of the lipidomics data from the different blood matrices; (**c**) The number of detectable lipid molecular species in different lipid classes; (**d**) Lipid class percentage distribution of total lipids in different blood matrices; (**e**) Reproducibility of lipid classes for different blood matrices (n = 8). **c** and **d** were run using one-way ANOVA with Dunnett’s post hoc test against DBS for each lipid class with *p-value < 0.05, **p-value < 0.01, and ***p-value < 0.001. PC, phosphatidylcholine; PE, phosphatidylethanolamine; PS, phosphatidylserine; SM, sphingomyelin; PI, phosphatidylinositol; PG, phosphatidylglycerol; PA, phosphatidic acid; TAG, triacylglyceride; DAG, diacylglyceride; Cer, ceramide; AC, acylcarnitines; Glyco, glycolipids; CoQ10, Coenzyme Q

To investigate the difference between DBS and other blood matrices, four blood matrices were assessed in this study: 1) whole blood (venous drawn), 2) plasma, 3) serum, and 4) finger pricked blood, the direct DBS. All samples were spotted onto Whatman 903™ filter paper cards (Fig. 1a). After 3 h, the spots were uniformly punched out and extracted using an automatic lipid extraction protocol using the Hamilton robotic system. Shotgun lipidomic analysis was performed using a customized high-resolution Triple-TOF system with data independent acquisition-MS/MS^ALL^ strategy [28]. Identification of lipid molecular species was based on the molecular weight, specific fragments, and a customized lipid database. Quantification was carried out by comparing the peak area of molecular species to the internal standard within a linear dynamic range. Appropriate dilution was performed to ensure the data was within the linear dynamic range and to minimize the aggregation of lipids in the solution. PCA analysis for different blood matrices (Fig. 1b) demonstrated similarity between the DBS and the whole blood, which is clearly separated from the plasma and serum. More than 1,200 lipid molecular species were determined in each blood matrix (Fig. 1c). Furthermore, the lipid class distribution (Fig. 1d) provided consistent results demonstrating finger prick DBS was analogous to whole blood samples, as they both contained more phospholipids but less triacylglyceride (TAG) than plasma and serum. Since the variation of single lipid molecular species is dependent on the concentration of the species and the coefficient of variation (CV) increases as the concentration decreases due to interference of background noise [34], we use total content of each lipid class to investigate the stability of lipids across all the blood matrices. The CV of each lipid class (including lyso molecular species) was used to assess the reproducibility for all four blood matrices (Fig. 1e). With the exception of phosphatidylinositol (PI) identified in whole blood sample, the CV for all the other lipid classes in various matrices examined were below 25%, which is acceptable for omics biomarker discovery studies [34].

### Short-term stability of DBS

**Fig. 2:**
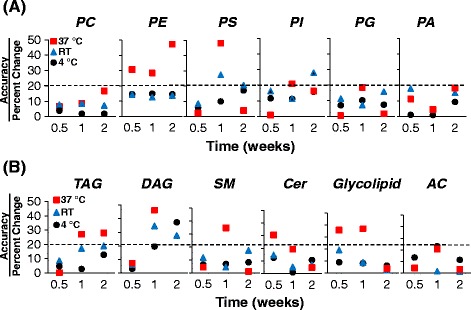
Assessment of short-term stability of dried blood spots (DBS)-MS/MS^ALL^ Lipidomics. (**a**) Phospholipids; (**b**) Glycerides, glycolipids, and lipid metabolites. PC, phosphatidylcholine; PE, phosphatidylethanolamine; PS, phosphatidylserine; SM, sphingomyelin; PI, phosphatidylinositol; PG, phosphatidylglycerol; PA, phosphatidic acid; TAG, triacylglyceride; DAG, diacylglyceride; Cer, ceramide; AC, acylcarnitines; Glyco, glycolipids; CoQ, Coenzyme Q

One of the advantages of DBS samples is improved analyte stability over a wide range of temperatures during storage and transportation [5, 6]. Here, we stored DBS samples at 4 °C, room temperature (RT), and 37 °C for 3 days, 1 week, and 2 weeks. The stability was determined by comparing the concentrations of each lipid class against those of the control samples (day 0) (Fig. 2). For almost all of phospholipids and glycolipids classes, relative change was less than 20% when samples were stored at 4 °C or RT for up to 2 weeks, except for diacylglyceride (DAG). In contrast, storing at a temperature of 37 °C impacted the stability for the vast majority of lipid classes, as expected, and increased the variability.

### Postprandial triglyceride profiling by DBS-MS/MS^ALL^ lipidomics

**Fig. 3:**
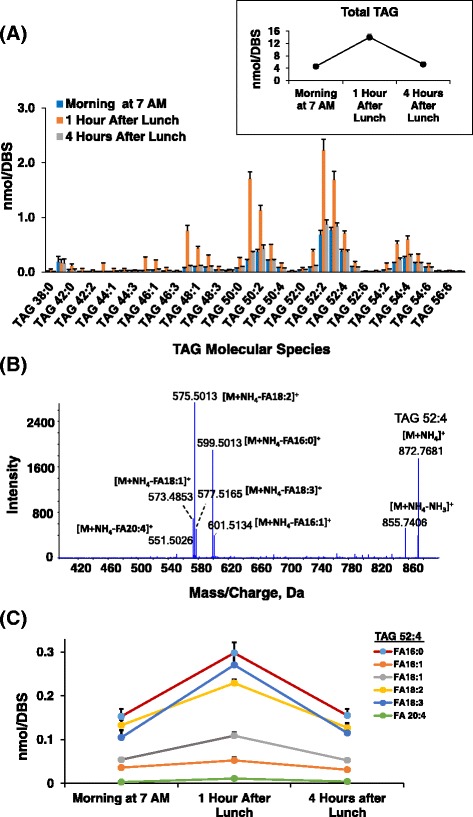
Postprandial triacylglyceride (TAG) profile using blood spots (DBS)-MS/MS^ALL^ Lipidomics. (**a**) Postprandial profile of TAG molecular species (insert: total TAG levels); (**b**) Identification of TAG 52:4 based on MS/MS fragmentation spectrum; (**c**) Temporal changes of TAG 52:4 with fatty acid components

Dynamic assessment of triglyceride content and molecular species in real-time without the need for clinical sample collection represents a promising approach to monitor triglyceride homeostasis in response to diet and clearance. Using the DBS-MS/MS^ALL^ lipidomics platform, we can measure not only the postprandial total TAG responses (Insert in Fig. 3a), but also identify and quantitate the level of TAG molecular species (Fig. 3a). For a designated TAG molecular species representative (e.g. TAG 52:4), through the MS/MS fragmentation and high-resolution capabilities (Fig. 3b), we identified the fatty acid components of TAG molecular species, and demonstrated the unique dynamic measurement of fatty acid building blocks during the course of the day for an individual molecular species (Fig. 3c).

### Temporal profiling of lipid species over time in a day using DBS-MS/MS^ALL^ shotgun lipidomics

**Fig. 4:**
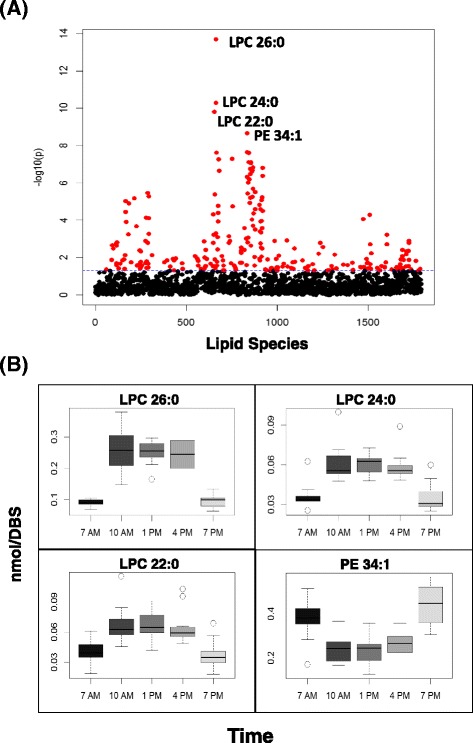
Temporal changes of lipid species over hours within a day. (**a**) The scatter plot of negative log_10_ (p) vs the lipid species, analyzed by repeated one-way ANOVA (subject = 12, 5 time points); (**b**) The profiles of the molecular species with the lowest p values over time. The DBS samples were collected at 7 AM, 10 AM, 1 PM, 4 PM, and 7 PM. ﻿ *LPC* lysophosphatidylcholine, *PE* phosphatidylethanolamine

To study the temporal fluctuation of lipid species, 12 volunteers were recruited and DBS collection was performed. During the day, 5 time points (7 AM, 10 AM, 1 PM, 4 PM, and 7 PM) were collected, extracted, and analyzed using DBS-MS/MS^ALL^ lipidomics. The negative log_10_ (p-value) was plotted against each individual lipid molecular species (Fig. 4a) to search for the most dynamic lipid molecules that are altered over the course of a day. The profile of lipid species with the smallest p-values, i.e. those that were significantly altered overtime are illustrated in Fig. 4b, which depicts lysophosphatidylcholine (LPC) 26:0, LPC 24:0, and LPC 22:0 concentration change is greatest between 10 AM and 4 PM and lowest between 7 PM and 7 AM, whereas phosphatidylethanolamine (PE) 34:1 levels decreased between 10 AM and 4 PM and increased between 7 AM and 7 PM. For LPC 26:0 and LPC 24:0, MS/MS spectra and fragmentation patterns of LPC 26:0 and LPC 24:0 were provided to support the identification (Additional file 3: Figure S1). In addition, we performed the same analysis for lipid classes (Additional file 4: Figure S2) and found that lipid classes, PE, sphingomyelin (SM), and Coenzyme Q (CoQ), varied significantly over the course of time within a day (p value < 0.05). Thus, given the dynamic changes in these lipid species and classes over the course of the day these results demonstrate that these lipid entities may be affected by circadian rhythms or dietary interactions and should be viewed with caution with respect to biomarker development.

### Temporal changes of fasted samples collected over 5 days using DBS-MS/MS^ALL^ lipidomics

**Fig. 5:**
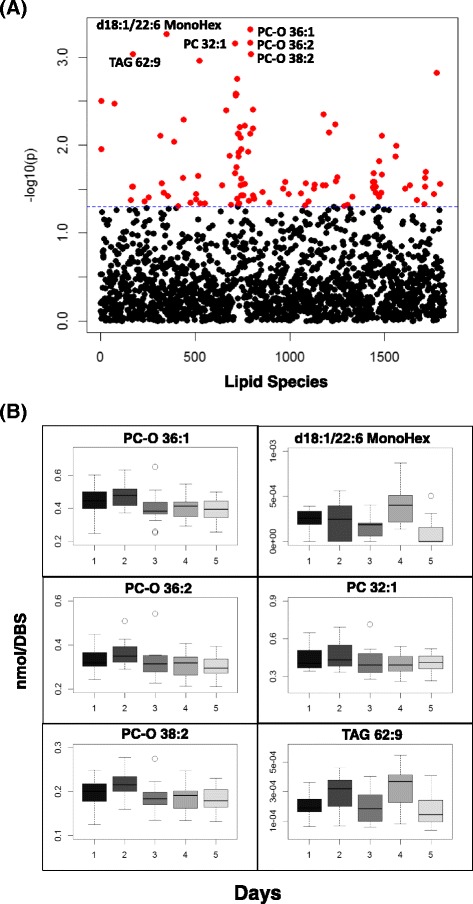
Temporal fluctuation of lipid species over days. (**a**) The scatter plot of negative log_10_ (p) vs the lipid species, generated by the repeated one way ANOVA analysis (subject = 16, 5 days); (**b**) The profiles of the molecular species with the lowest p values over time. The DBS samples were collected in the 5 successive morning after overnight fasting. *MonoHex* monohexose, *PC* phosphatidylcholine, *TAG* triacylglyceride

To assess the intrinsic day to day variation of the lipid molecular species using DBS MS/MS^ALL^ shotgun lipidomics analysis, sixteen healthy volunteers were recruited and DBS were collected over the course of 5 successive mornings after overnight fasting. The negative log_10_ (p-value) are plotted for each individual lipid molecular species (Fig. 5a). The profile of lipid species with the smallest p-values that are affected over time is illustrated in Fig. 5b. For lipid classes, only phosphatidylcholine (PC) showed significant changes across the week (Additional file 5: Figure S3).

**Fig. 6:**
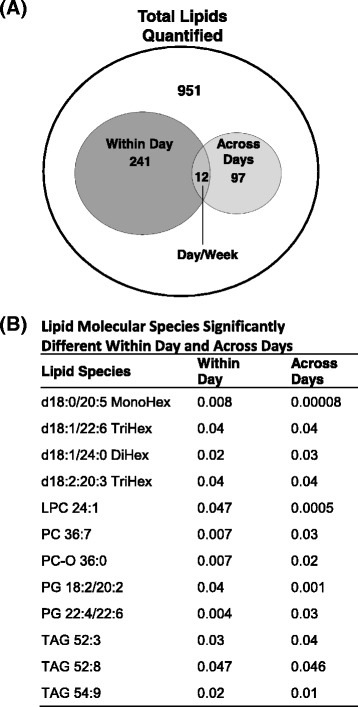
**a** Venn diagram of lipid molecular species significantly changed (p values < 0.05) within a day and across days; **b** Lipid species significantly changed within a day and across days. MonoHex, monohexose; BiHex, bihexoses; TriHex, trihexoses; PC, phosphatidylcholine; LPC, lysophosphatidylcholine; PG, phosphatidylglycerol; TAG, triacylglyceride

Across the different time scales we identified 253 lipids species that are significantly altered over the course of hours within the day (p values <0.05), which accounts for 19% of the total lipids detected, while 109 lipid molecules (8% of total measured lipids) were altered over the course of 5 days (Fig. 6a). Alterations of 12 species were found to overlap across both time scales (hours within a day and days across a week), which include multiple lipid classes, such as glycolipid, PC, phosphatidylglycerol (PG), and TAG (Fig. 6b).

## Discussion

Dried blood spot (DBS) analysis is a convenient way to collect blood samples with several advantages over conventional blood collection methods. DBS has gained popularity in fields such as newborn screening, preclinical studies, and therapeutic drug monitoring [2, 4, 7, 8]. DBS coupled with LC-MS/MS system provides the capacity to analyze samples in a high throughput manner once coupled to robust analytical methods. Lipidomics analysis of whole blood, which is comprised of thousands of diverse lipid molecular species, is directly linked to an individual’s physiological, nutritional and health status [14, 35]. In this study, we combined DBS collection with high-resolution MS/MS^ALL^ shotgun lipidomics analysis to analyze the blood lipidome. We demonstrate in one DBS spot, several lipid classes and more than 1,200 lipid species were identified and quantified.

Direct infusion-based MS shotgun lipidomics provides comprehensive profiling and quantitation of lipid species from organic extracts of biological samples without the LC column separation [22]. Conventionally, numerous tandem MS strategies have to be applied for different lipid classes, such as precursor ion scanning and neutral loss scanning, and it requires the pre-selection of target lipids and fragmentations. On the other hand, the high-resolution MS/MS^ALL^ method, which utilizes DIA analysis, does not require any pre-selection and records all information in high resolution MS/MS spectra, thus allowing for data interpretation following the analysis [28]. It is noteworthy to mention that the MS/MS^ALL^ approach employs 1 m/z isolation window instead of the wider windows, because the wider isolation windows result in the disconnection between the fragment ions and precursors, complicating the analysis of the acquired data [25]. Using the 1 Da isolation window not only allows us to collect all the fragments from the precursors, but maintains the relationship between the fragments and the precursor ions, which facilitates the identification of lipid molecular species through data processing. Collectively, the MS/MS^All^ approach is bias-free and delivers the high specificity with informative production ion spectra and high sensitivity with the limit of quantitation (LOQ) as low as the sub nM or nM range for most of lipids, and offers a solution for measuring the lipidomes in diverse clinical samples using a high throughput approach that enables application of lipidomics in broader population-based analysis.

The era of precision medicine is now taking center stage, accelerating the stratification of patients based on their molecular signatures [36]. Genomics alone, however, will not be able to selectively define all patient populations prone to a disease or therapeutic response. Proteomic, metabolomic, and lipidomic workflows are emerging dynamic “omics” technology, which can be complementary to genomics. Thus, illuminating the complexity of environmental exposure and phenotype of health as well as disease states [37, 38].

In this study the postprandial TAG molecular species and the entire lipidome identified by the DBS-MS/MS^ALL^ lipidomics platform delivered important insight that enables the potential for personal lipidomics profiling to monitor physiological changes, which could be applied to the diagnosis and presence of disease. It is notable that there are higher variations of lipid molecular species within the day than within the week (across days) (Fig. 6a), which indicates that internal factors (such as circadian rhythms), and external factors (i.e. diet) have a profound effect on the blood lipid profiles. This is not surprising given that several lipid classes and species have been demonstrated to show diurnal variation in animal and human studies [39]. Thus, these data implicate the need to consider time of day/circadian rhythm when assessing specific lipid species for comparisons across individuals. However, the consistency of the lipid profiles across days implies that fasted blood samples are more stable across days when assessed at the same time among various individuals. The lipid molecular signatures from the temporal changes of lipid species over different time periods (hours vs days) using DBS-MS/MS^ALL^ lipidomics demonstrates the feasibility to use this technique for biomarker discovery in population-based studies and highlights the innate temporal changes of the lipidome in diverse individuals. Taken together, the DBS-MS/MS^ALL^ lipidomics platform, with its simple blood collection and high throughput capacity could be applied for future large-scale population based lipidome profiling.

As a less invasive sampling method DBS offers a simple collection protocol, requires a significantly smaller blood volume than venous sampling, and is easy to store and transfer, which thereby reduces the infection risk to various pathogens [5, 6, 40]. DBS coupled with LC-MS/MS provides a highly sensitive and selective method for quantitative analysis of small molecules, lipids, peptides, and proteins [8, 11, 41]. The challenges for developing DBS-LC-MS/MS methods include the lack of extensive references for most molecules using DBS for blood sampling, the discrepancy among blood and plasma/serum, and the possible interaction of blood and/or analytes with the matrix of a DBS card [40, 42], which were addressed by some of the experiments reported in this manuscript.

## Conclusions

In summary, here in we developed a novel DBS-MS/MS^ALL^ shotgun lipidomics workflow to profile the human blood lipidome. In a single DBS spot, multiple lipid classes and more than 1,200 lipid species were identified and quantified. The application of this method to the postprandial lipidomics profiles and the temporal changes of lipids over time demonstrated the potential for use in preclinical and clinical studies, as well as for population-based health studies. Analysis of an individual’s lipidome using this novel platform provides a valuable approach for monitoring health status in real-time and provides a comprehensive profile of a person’s lipid homeostatic state.

## Additional files

Additional file 1: Table S1. Internal standards for each lipid class (PDF 7 kb)
Additional file 2: Table S2. Extraction method repeatability, recovery, and limit of quantification (LOQ) for each lipid internal standard added to DBS samples (PDF 90 kb)
Additional file 3: Figure S1. Identification LPC 24:0 and LPC 26:0 from DBS by high resolution MS/MS. (A) MS/MS spectrum and fragmentation pattern of LPC 24:0; (B) MS/MS spectrum and fragmentation pattern of LPC 26:0. (PDF 89 kb)
Additional file 4: Figure S2. Temporal changes of lipid classes over hours in a day. (A) The scatter plot of negative log_10_ (p) vs the lipid classes, generated by the repeated one way ANOVA analysis (subject = 12, 5 time points); (B) The profiles of the lipid classes with a p value of less than 0.05. The DBS samples were collected at 7 AM, 10 AM, 1 PM, 4 PM, and 7 PM. (PDF 24 kb)
Additional file 5: Figure S3. Temporal fluctuation of lipid classes over days. (A) The scatter plot of negative log_10_ (p) vs the lipid classes, generated by the repeated one way ANOVA analysis (subject = 16, 5 days); (B) The profiles of the lipid class with a p values of less than 0.05 over time. The DBS samples were collected in the 5 successive morning after overnight fast. (PDF 16 kb)

## Abbreviations

AC: Acylcarnitine
ANOVA: Analysis of variance
APCI: Atmospheric-pressure chemical ionization
BiHex: Bihexoses
Cer: Ceramide
CoQ: Coenzyme Q
CUR: Curtain gas
CV: Coefficient of variation
DAG: Diacylglyceride
DBS: Dried blood spot
DIA: Data-independent acquisition
ESI-MS: Electrospray ionization mass spectrometry
FA: Fatty acid
Glyco: Glycolipids
HPLC: High performance liquid chromatography
LC: Liquid chromatography
LC/MS: Liquid chromatography/mass spectrometry
LiCl: Lithium chloride
LPC: Lysophosphatidylcholine
MeOH: Methanol
min: Minute
MonoHex: Monohexose
ms: Millisecond
PA: Phosphatidic acid
PC: Phosphatidylcholine
PE: Phosphatidylethanolamine
PG: Phosphatidylglycerol
PI: Phosphatidylinositol
PS: Phosphatidylserine
SD: Standard deviation
SM: Sphingomyelin
TAG: Triacylglyceride
TriHex: Trihexoses.
