# Supplementary material for: Dynamic and temporal assessment of human dried blood spot MS/MSALL shotgun lipidomics analysis
Source: Nutr Metab (Lond). 2017 Mar 20;14:28. doi: 10.1186/s12986-017-0182-6 (PMC5360027; doi:10.1186/s12986-017-0182-6)
Supplement: Additional file 1: Table S1. — Internal standards for each lipid class (PDF 7 kb) [file 12986_2017_182_MOESM1_ESM.pdf]

Supplemental Table 1. Internal standards for each lipid class

Internal Standards[a]	Concentration (nmol/ DBS spot)
PC 14:1/14:1	11.00
LPC 17:0	2.20
PE 16:1/16:1	0.55
LPE 14:0	0.11
PG 15:0/15:0	0.33
PS 14:0/14:0	0.33
PA 12:0/12:0	0.44
TAG 17:1/17:1/17:1	6.60
DAG 17:1/17:1	1.65
SM 12:0	1.10
Cer 17:0	0.066
$^{13}C_4$-AC 16:0	0.011
CBS 15:0	0.025

[a] PC, phosphatidylcholine; PE, phosphatidylethanolamine; PS, phosphatidylserine; SM, sphingomyelin; PI, phosphatidylinositol; PG, phosphatidylglycerol; PA, phosphatidic acid; TAG, triacylglyceride; DAG, diacylglyceride; Cer, ceramide; AC, acylcarnitine; LPC, lysophosphatidylcholine; LPE, lysophosphatidylethanolamine; CBS, cerebroside.
