# Supplementary material for: Dynamic and temporal assessment of human dried blood spot MS/MSALL shotgun lipidomics analysis
Source: Nutr Metab (Lond). 2017 Mar 20;14:28. doi: 10.1186/s12986-017-0182-6 (PMC5360027; doi:10.1186/s12986-017-0182-6)
Supplement: Additional file 2: Table S2. — Extraction method repeatability, recovery, and limit of quantification (LOQ) for each lipid internal standard added to DBS samples (PDF 90 kb) [file 12986_2017_182_MOESM2_ESM.pdf]

Supplemental Table 2. Extraction method repeatability, recovery, and limit of quantification (LOQ) for each lipid internal standard added to DBS samples

Analyte[a]	Repeatability		Recovery of added analytes[b], %	LOQ (nM)[c]
	Peak Intensity[b]	CV (%)		
PC 14:1/14:1	21753333 ± 555008	2.6	105.2 ± 2.7	0.16
PE 16:1/16:1	884750 ± 23210	2.6	52.4 ± 1.4	0.81
LPE 14:0	35220 ± 4256	12.1	47.6 ± 5.8	1.61
LPC 17:0	6827833 ± 482250	7.1	78.0 ± 5.5	0.32
TAG 17:1/17:1/17:1	12831667 ± 661331	5.2	104.8 ± 5.4	0.10
DAG 17:1/17:1	1464167 ± 127033	6.4	51.7 ± 4.5	0.24
CBS 15:0	61342 ± 4049	6.6	59.4 ± 3.9	0.37
SM 12:0	44387 ± 3799	8.6	114.0 ± 9.8	0.16
$^{13}C_4$ -AC -16:0	77268 ± 4157	5.4	70.3 ± 3.8	1.62
Cer 17:0	197133 ± 15760	8.0	68.8 ± 5.5	0.97
PG 15:0/15:0	1267200 ± 123808	10.1	96.1 ± 9.7	0.49
PS 14:0/14:0	166300 ± 14012	8.4	117.1 ± 9.9	0.49
PA 12:0/12:0	62825 ± 3185	5.1	55.3 ± 2.8	0.65

[a] Abbreviations are the same as Supplemental Table 1.

[b] Data are presented as Mean ± SD (n=3)

c LOQ, limit of quantification
