# Supplementary figures and images for: Dynamic and temporal assessment of human dried blood spot MS/MSALL shotgun lipidomics analysis
Source: Nutr Metab (Lond). 2017 Mar 20;14:28. doi: 10.1186/s12986-017-0182-6 (PMC5360027; doi:10.1186/s12986-017-0182-6)

# Supplemental Figure 1

(A)

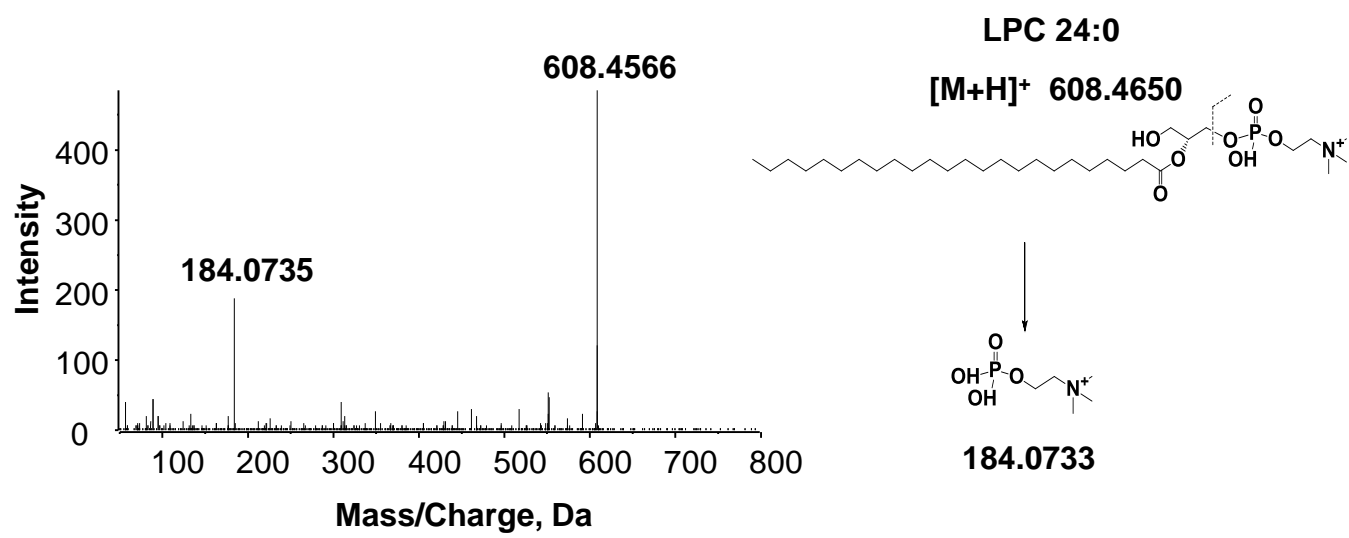

(B)

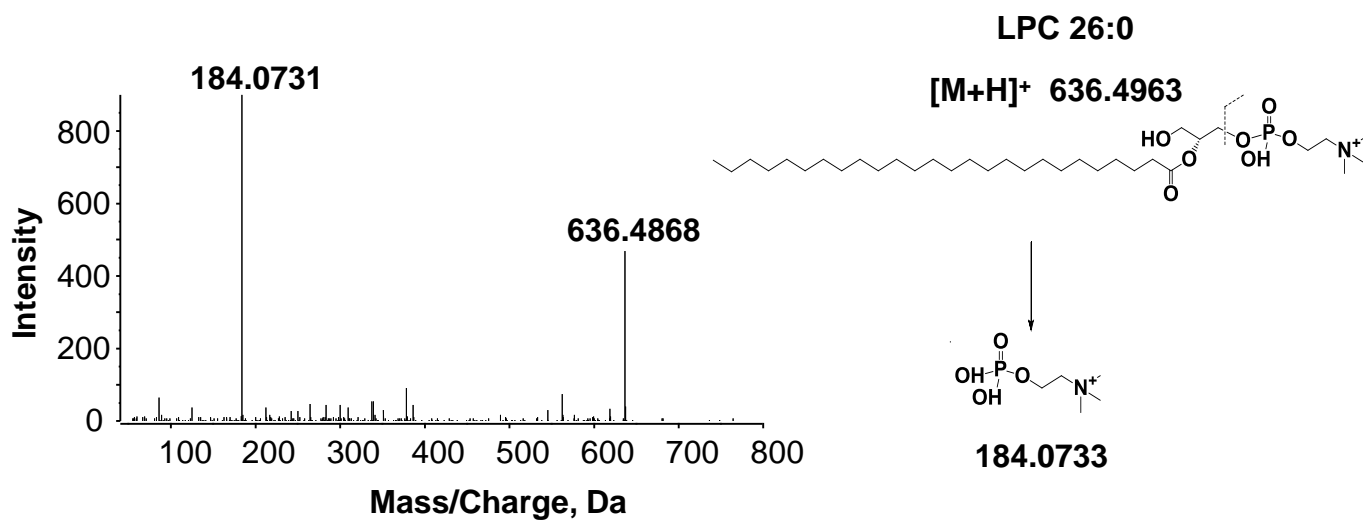

Supplement: Additional file 3: Figure S1. — Identification LPC 24:0 and LPC 26:0 from DBS by high resolution MS/MS. (A) MS/MS spectrum and fragmentation pattern of LPC 24:0; (B) MS/MS spectrum and fragmentation pattern of LPC 26:0. (PDF 89 kb) [file 12986_2017_182_MOESM3_ESM.pdf]

Supplemental Figure 2

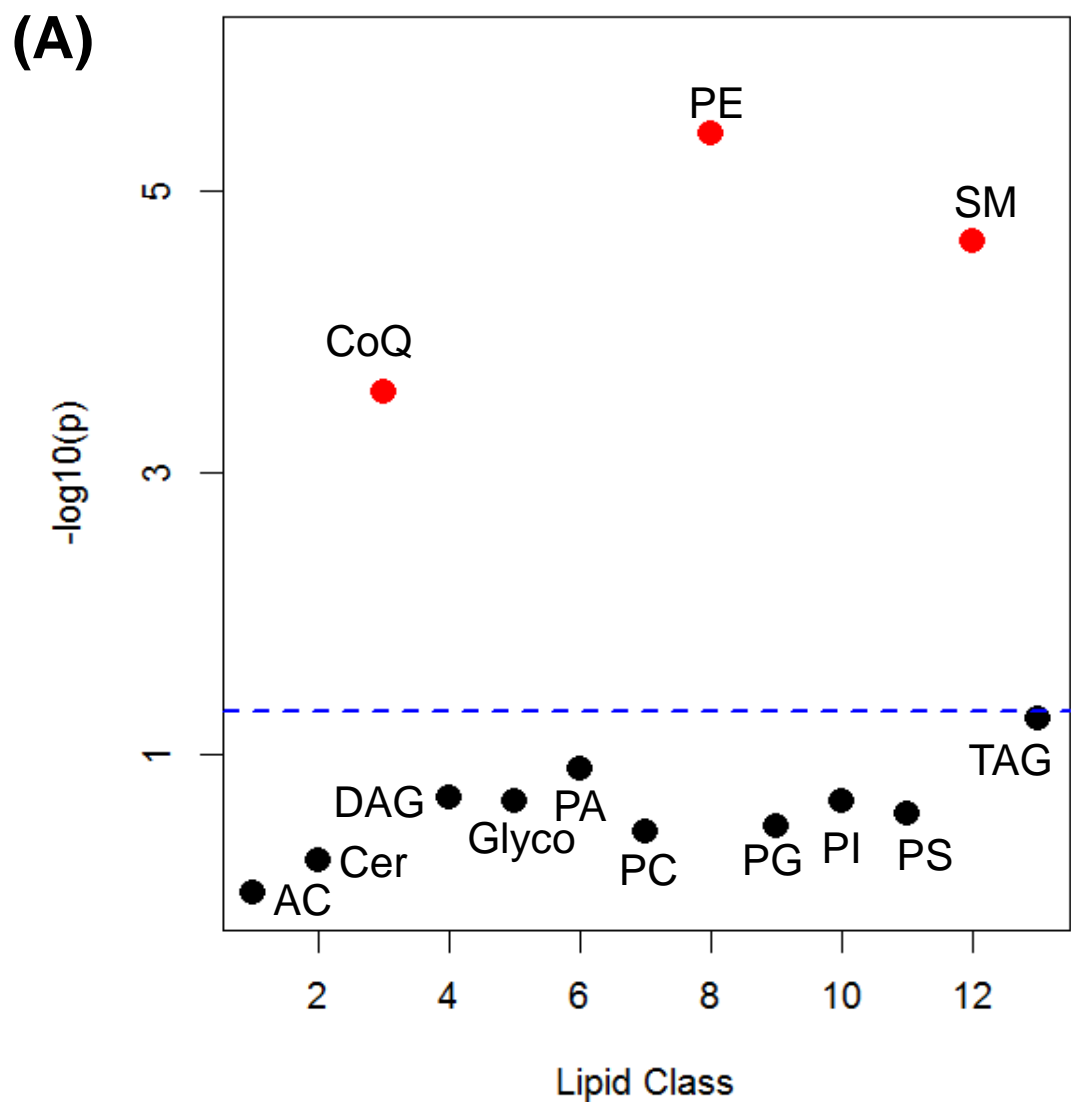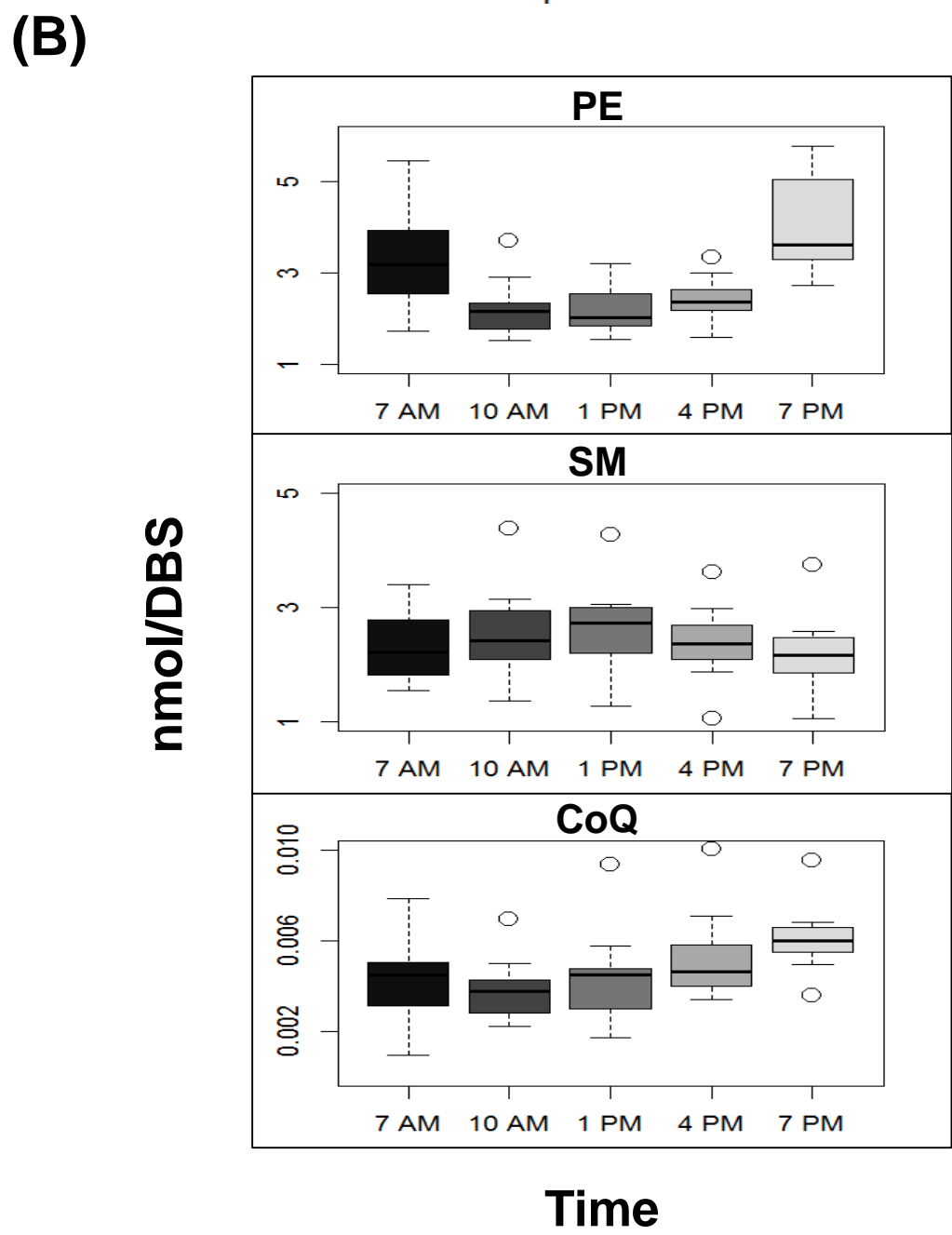

Supplement: Additional file 4: Figure S2. — Temporal changes of lipid classes over hours in a day. (A) The scatter plot of negative log10 (p) vs the lipid classes, generated by the repeated one way ANOVA analysis (subject = 12, 5 time points); (B) The profiles of the lipid classes with a p value of less than 0.05. The DBS samples were collected at 7 AM, 10 AM, 1 PM, 4 PM, and 7 PM. (PDF 24 kb) [file 12986_2017_182_MOESM4_ESM.pdf]

Supplemental Figure 3

(A)

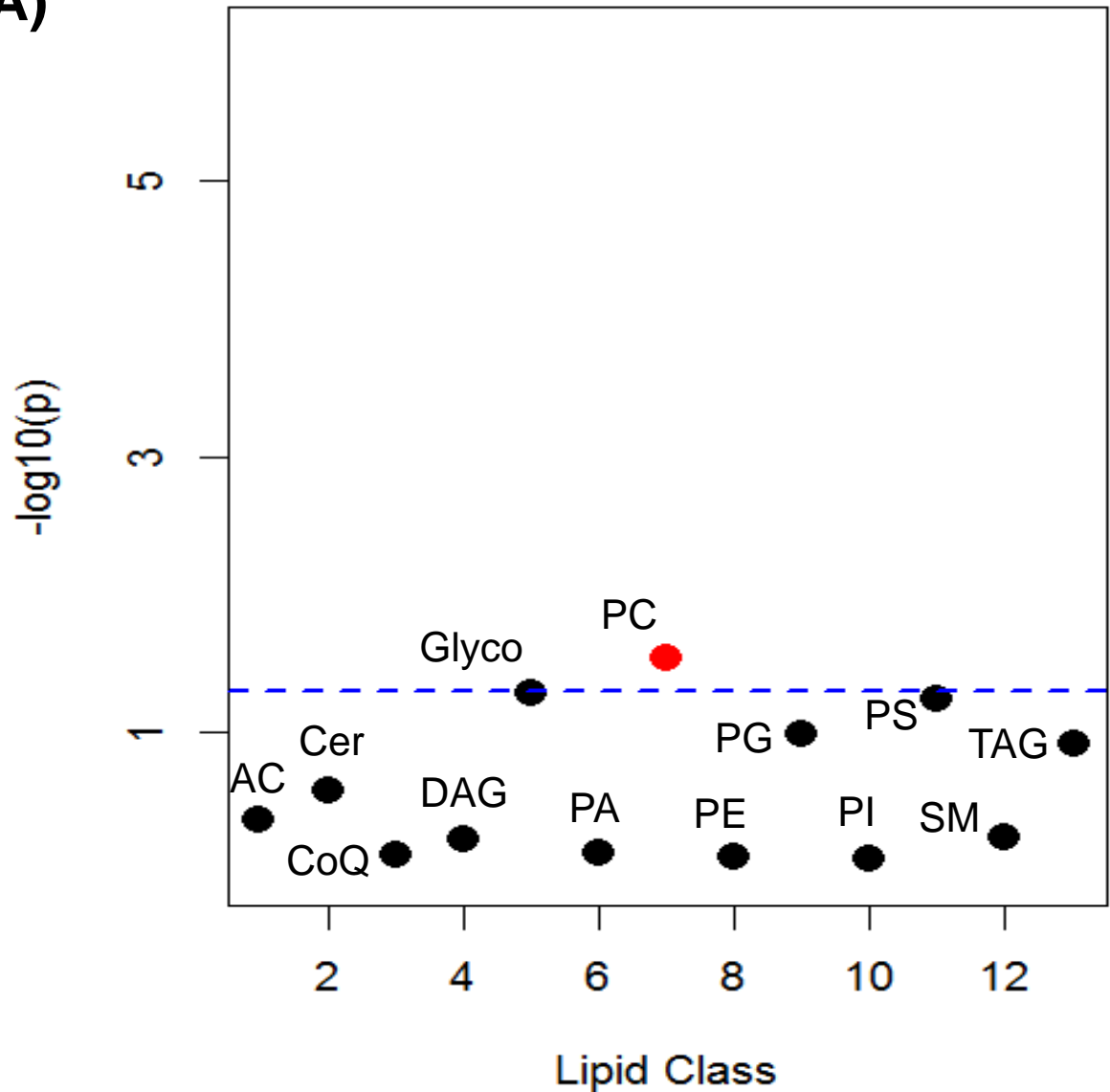

(B)

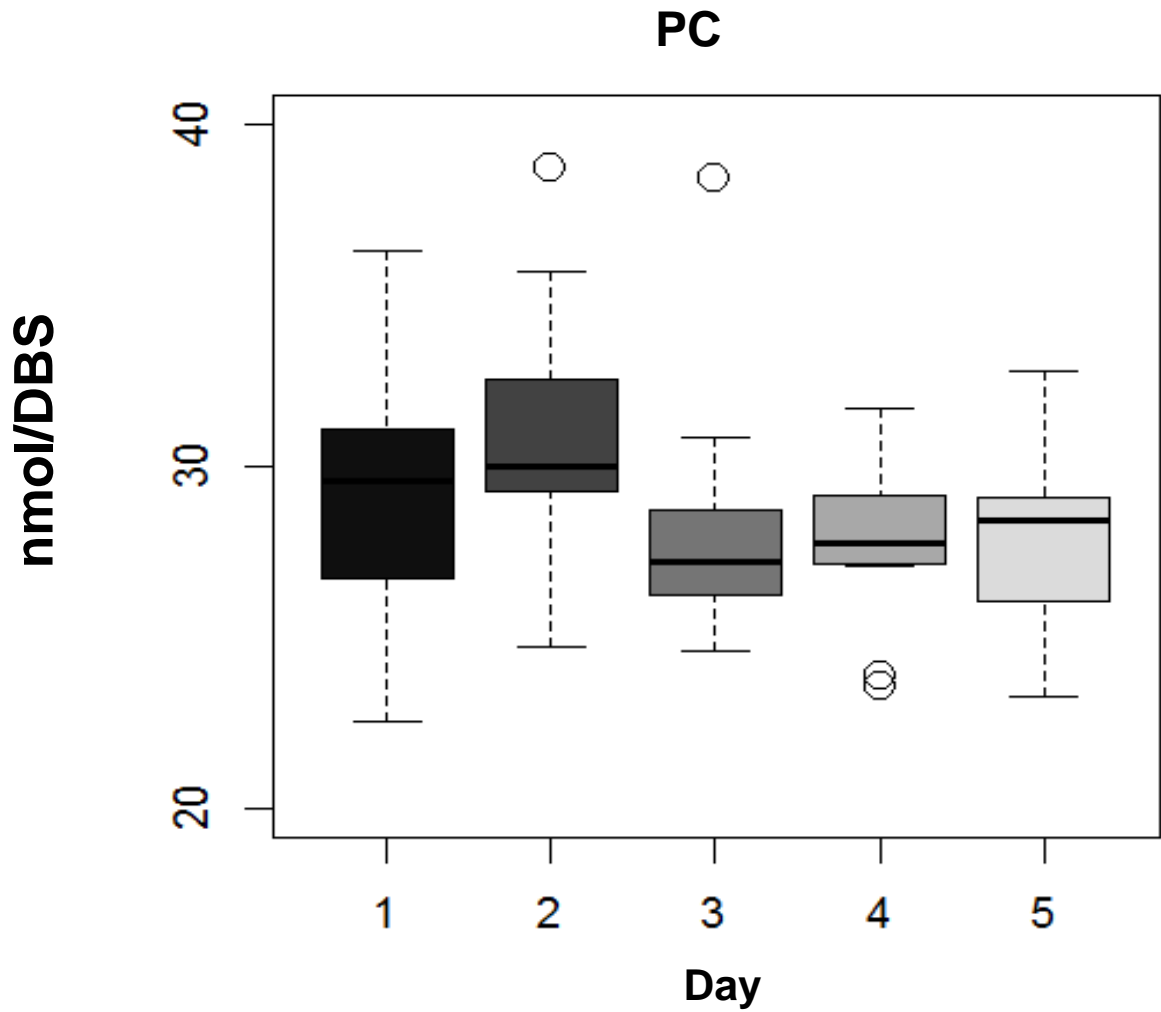

Supplement: Additional file 5: Figure S3. — Temporal fluctuation of lipid classes over days. (A) The scatter plot of negative log10 (p) vs the lipid classes, generated by the repeated one way ANOVA analysis (subject = 16, 5 days); (B) The profiles of the lipid class with a p values of less than 0.05 over time. The DBS samples were collected in the 5 successive morning after overnight fast. (PDF 16 kb) [file 12986_2017_182_MOESM5_ESM.pdf]
